# Modelling Influence on Bending Behaviour Simulation of the Poly(Lactic Acid) Structures, 3D Printed

**DOI:** 10.3390/polym15040960

**Published:** 2023-02-15

**Authors:** Dorin-Ioan Catana, Denisa-Iulia Brus, Mihai-Alin Pop

**Affiliations:** 1 Department of Materials Engineering and Welding, Transilvania University of Brasov, 500036 Brasov, Romania; 2 Interdisciplinary Doctoral School, Transilvania University of Brasov, 500036 Brasov, Romania; 3 Department of Materials Science, Transilvania University of Brasov, 500036 Brasov, Romania

**Keywords:** additive manufacturing, poly(lactic acid), bending, simulation

## Abstract

The paper presents the influence of the loading modelling on the simulation process results of the bending behaviour for 3D printed structures. The study is done on structures having different geometries of the cross section, and the type of structure is bar or tube. The materials used for 3D printing are poly(lactic) acid and poly(lactic acid) mixed with glass fibres. The simulation was carried out both based on a simple modelling (schematization) of the bending loading and a complex one. The complex modelling reproduces the bending of 3D printed structures more accurately but is also more time-consuming for the computer-aided design stage. Analysis of the study results shows that in terms of the Von Mises stresses determined by simulation, they are in line with those of the tests but with a slight advantage for the complex modelling compared to the simple one. In terms of deformations, the simulation introduces errors compared to the test results, but the source of the errors is the high elasticity of some 3D printed structures. The study also shows that the high elasticity is due to both the shape of the structure cross section and its arrangement during the bending test.

## 1. Introduction

Influences on materials manufacturing processes show that they have had a continuous evolution and have adapted to the needs of the human species. Currently, there are manufacturing processes that have existed since the Stone Age, while others have been implemented more recently, namely in the twentieth or even twenty-first century. All have in common that their development has been made possible by the intuition, ability to innovate, creativity and determination of certain people. In addition to the mentioned abilities, a substantial contribution was brought by the progress of the exact sciences (mathematics, physics, mechanics) without which the manufacturing processes would not have reached the current stage. The second half of the twentieth century was marked by an exponential increase in computing power, based on the progress of electronics and software.

The combination of the principles of classical processing with the performance and capabilities of manufacturing equipment but also with 3D design software has made possible the emergence of a new technology, namely additive manufacturing, also known as 3D printing. A new technology involves conducting studies to solve problems related to the materials that can be processed but also the parameters with which they can be processed. Through their approaches, researchers are trying to provide acceptable solutions to the problems reported by those who process 3D printed structures. In the case of a three-point bending fixture, the flexural testing for a 3D printed poly(lactic acid) (known as PLA) specimen showed that the 0° raster orientation had the greatest value of ultimate bending stress (102 MPa) [1]. The most frequently used solution is finite element analysis (FEA) or finite element modelling (FEM). The analysis with a finite element or in short the simulation process (or simulation) is a tool available for researchers that allows them to accelerate the obtaining of the correct solutions to the analysed problems, with low costs and in a short time. Like any other technology, simulation is based on the physical, mechanical and technological properties of 3D printed parts (structures). The large number of materials and technologies of additive processing makes difficult the mission of researchers to find the optimal manufacturing parameters in order to maximize the performance of 3D printed structures. For example, a study shows that 3D printed PLA structures had superior properties (the ultimate tensile strength) in comparison with 3D printed, bronze-filled PLA [2].

Now, many researchers are focused on improving the behaviour of the poly(lactic acid)-PLA. The simulation process was used to study the interactions in the mandibular joint. The models of maxilla mandible and disc were created by 3D printing of the PLA. The conclusion is that the difference between the experiments and FEA is less than 5% in the case of horizontal and vertical strains [3]. Finite element analysis can be used to indicate the performance of body parts manufactured by 3D printing. For PLA parts, the safe conditions were obtained when printing took place in certain conditions: an infill density of 75% and a honeycomb pattern geometry [4]. By implication of FEA in the design and 3D printing process, the prosthetic foot can be reduced up to 62% [5]. For the three-point bending testing, in the case of PLA, the difference between FEA results and the ones experimentally determined is around 9% when the specimen is printed with 100% infill density. The difference increases when the infill density is 50% [6]. Regarding tensile strength, finite element analysis reveals that the major contribution to mechanical properties of 3D printed parts is made by shell thickness and type of infill, followed by lattice design [7]. FEA also helped the researchers to determine the Poisson’s ratio at the tension test with a small error, in comparison with the value obtained by experiments [8]. After creating a model, this was evaluated by computational simulation to show how it responds to a four-point bending test.

The FEA utilization provides results that coincide with the experimental values in the case of 3D printed wood–PLA [9]. When the specimen length increases, the error of Young’s modulus decreases. For a span of 70 mm, the error is around 2.5%, but for a span of 350 mm it can be zero. Moreover, the ratio between span and height (thickness) should be greater than 50 for a width of 25 mm [10]. Many studies reveal that the maximum tensile strength (Von Mises stress) is obtained at a printing angle of 45°/−45° and under the assumption that the specimens have isotropic properties [11]. For the 3D printed parts, the highest strength and ductile behaviour can be obtained in the longitudinal direction, while the transversal direction exhibits a fragile behaviour at the yielding point and is weaker [12]. Orthotropic material hypothesis and transversely isotropic hypothesis under plane stress state are suitable for mechanical analyses of 3D printed PLA. The value of the Young’s modulus depends on printing angle and layer thickness (increase with increase of printing angle and decrease with decrease of the layer thickness) [13]. Moreover, for the 3D printed PLA, experimental simulation or investigation was used to understand the behaviour of the uniaxial tensile response at three temperatures [14] or to establish the deposited filament properties when nozzle temperature and velocity were changed [15].

The aim of this paper is to study how the precise modelling of the stress influences the simulation results in the bending behaviour of the 3D printed PLA structures. Previous studies [16,17] show that in the case of the bending simulation of additively manufactured PLA, important errors have been recorded in some cases, especially for the deformation values. On the other hand, the mentioned research shows that in the case of Von Mises stresses, the simulation results show errors of ±7% compared to the results recorded by tests. It is important to determine whether these errors are due to the properties of additively manufactured structures or are of a different nature. It should be noted that due to the way that the additive manufacturing takes place, the properties of the filament will not be transferred to the manufactured parts (structures). In other words, the mechanical and technological properties of the 3D printed structures will not be identical to those of the filaments from which they were obtained.

Industry 4.0 includes all manufacturing phases of the products, starting from the idea and finishing with recycling. By using the software modules (CAD, CAE, CAM, FEA, PLM) the necessary time for the design and optimizing of the parts is significantly reduced.

## 2. Theoretical Considerations

The products are composed of several parts whose shape is often complicated and complex. Engineering models schematize them to certain simple forms, convenient for establishing computational relations, so that the schematization does not produce a divergence from the real phenomenon. The modelling (schematization) must not affect the way how the piece is stressed or its role in the assembly of the whole. In engineering, there are frequent cases in which the hypothesis of the rigid solid body is replaced with that of the deformable solid body. Under the action of external forces, the solid bodies are deformed, even if their value is reduced. Depending on their size, solid bodies can be grouped as follows:

- wires or bars that have a much larger dimension compared to the other two;
- plates that have two dimensions greater than the third;
- blocks that have all dimensions of the same order.

The structures obtained by additive manufacturing from PLA are of the following type: bars, plates, or blocks. In order to be able to establish computational relationships, the material strength (the strength of materials) implies the fulfilment of certain hypotheses relating to the material of the solid body and the state of stress to be met. The calculus relations deduced based on hypothesises were verified by the existence of engineering achievements and validate the fact that the adopted hypothesis reflect, with a good approximation, the reality. It is true that their use admits computational errors, but the latter are accepted in engineering. The main hypothesises are:

- the continuous environment hypothesis that admits that the structures are formed by a continuous environment that occupies the entire space delimited by volume;
- the hypothesis of homogeneity and isotropy which considers that the materials have the same properties in all points and in all directions;
- the hypothesis of the identity of the mechanical properties of the infinitely small element with those of the whole solid body;
- the hypothesis of perfect elasticity which considers that when the stress does not exceed certain limits, after its removal the solid body returns completely to its original shape and dimensions.

The hypothesises described are fulfilled by the 3D printed structures in PLA. Under the action of forces, the solid body deforms, and its internal points move. Depending on the number and nature of the connections, the displacements of the points of the solid body can be:

- kinematic displacements when the connections allow mechanical movements, the solid body changing its position in space;
- displacements produced by the deformation of the solid body when under the action of forces they change their dimensions and initial geometric shape.

**Figure 1:**
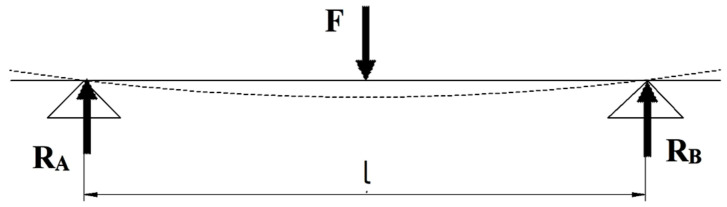
Simple modelling (SM) of bending stress.

**Figure 2:**
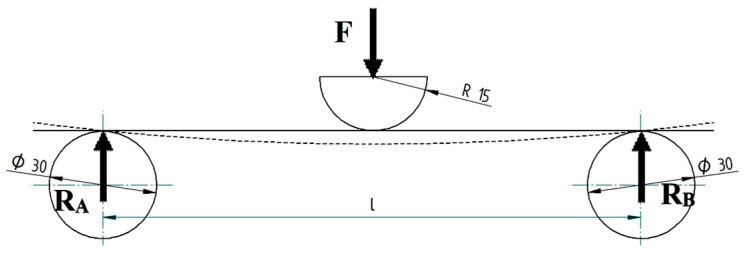
Complex modelling (CM) of bending stress.

Straight beams deform and bend during bending stress. The study of deformations aims to determine the shape of the beam under the action of forces or to establish the displacements produced near the calculation sections. The displacement of the cross sections (of the middle fibre) is called the maximum deflexion (arrow) and represents the ordinate *v* corresponding to an abscissa (v = f (x)). Figure 1 shows the simple modelling (SM) of the bending stress of a beam. It is observed that the transmission of the stress from the force applied to the beam is punctual (the contact between the two elements is a point). Moreover, the loading (stress) from the beam to the two supports is also transmitted punctually. This type of schematization was used in the process of simulating the 3D printed PLA structures stressed for bending. Figure 2 shows a new approach to the representation (much closer to reality) of the bending stress (complex modelling—CM). The loading force is applied to the specimen by anvil (loading nose/pin) which has a semi-cylindrical shape (diameter 30 mm and a length of 70 mm) and is in accordance with the shape of the respective element in the endowment of the universal test machine.

The supports (supporting pin) on which the stressed beam is placed are cylindrical, with a diameter of 30 mm and a length of 70 mm. A simple representation of the bending has become a much more complex one, but the modelling involves many more hours of design. In the case of complex schematization, for cylindrical specimens, the transfer from the anvil to the specimen is done in the same way as in the simple schematization because the element of anvil intersects with the element of specimen, that is, the intersection of two straight lines. The intersection of two lines is a point. The same goes for the transfer from the test tube to the supports. In the case of the specimens with a rectangular cross section, the stress shall be transmitted from the anvil to the specimen and from the specimen to the supports by means of straight lines. This is because the intersection between the element of anvil and the top surface of the test piece is a straight line. The same applies to the contact between the lower surface of the specimen and the supports. In conclusion, the contacts between the elements involved in the bending stress are no longer point-like but linear. The simulation process applied to this complex schematization (modelling) of the bending stress will determine the magnitude of the errors between the simulation and test results.

Information from previous research on simple schematization shows that in the case of deformations, there are sometimes large differences between the results of simulations and those of tests. In terms of Von Mises stresses, these errors have lower values. By appealing to the complex schematization of the bending stress, it is investigated whether the values of the deformations obtained by simulation are close to those recorded by tests.

## 3. Experiment Setup

In the study, two types of filaments based on poly(lactic acid) were used for 3D printing of structures. The first filament was poly(lactic acid) manufactured by Suntem3D, and the mechanical properties were: filament diameter 2.85 mm, tensile strength 1100 MPa (ASTM D882), modulus of elasticity 3310 MPa (AST MD882), bending modulus of elasticity 2392.5 MPa. The second filament was poly(lactic acid) mixed up to 20% with glass fibre (PLA-Glass manufactured by Filaticum) with the following mechanical properties: filament diameter 2.85 mm, maximum tensile strength 57 MPa (ASTM D638), tensile strength at yield 46 MPa (ASTM D638), tensile modulus 4.0 GPA (ASTM D638), tensile elongation 3.4% (ASTM D638). The complete technical characteristics of the filaments are presented in their technical data sheets. These filaments were used in the 3D printing of the specimens used in the bending test. The geometry of the cross sections and the dimensions are different so that the tests provide a clearer picture of the behaviour of the specimens in the bending test. The 3D printing was done on a CreatBot DX-3D double-nozzle printer. The printer specifications were:

- printing dimensions—300 × 250 × 300 mm;
- filament diameter—2.8–3.0 mm;
- layer resolution—0.2 mm;
- printing nozzle—0.2–0.8 mm;
- printing resolution—0.6 mm;
- printing volume—22.5 l.

The parameters of the printing process were:

- layer height—0.2 mm;
- printing temperature—210 °C;
- print speed—50 mm/s;
- printing angle (overhang angle for support)—45°;
- bed temperature—61 °C;
- infill—100% (the internal structure is solid); also, solid infill at top and bottom;
- infill overlap—10%;
- infill flow—110%.

Additively manufactured specimens are of the bar or pipe type. Bending tests have been carried out in accordance with ISO-178 sixth edition 2019-04. Specimen codes by geometry, size and type are presented in Table 1.

**Figure 3:**
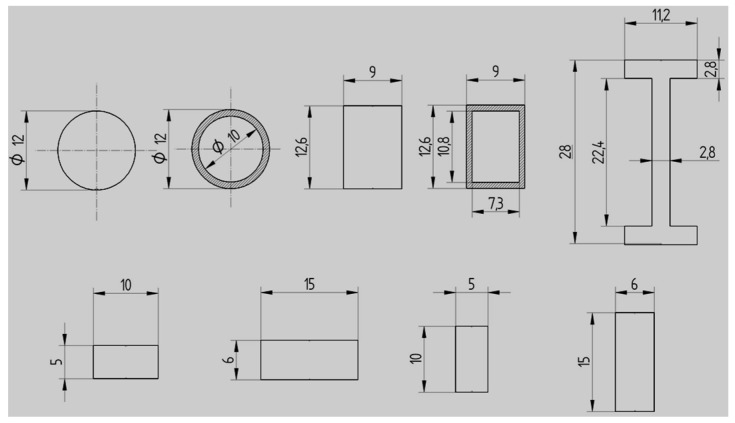
Dimensions and shapes of the specimens (ISO metric drawing standard).

The specimens’ dimensions and shapes are shown in Figure 3.

The study of references also shows that for the bending test researchers use specimens with different cross-section and length dimensions [18,19,20]. The equipment used for the bending test was a WDW-150S Universal testing machine. The bending tests conditions were:

- bending (loading) speed—10 mm/min;
- stress speed—10 MPa/s;
- supporting pin—cylindrical (diameter 30 mm, length 70 mm);
- test force —between 0.1 and 150 kN
- loading nose/anvil—semi-cylindrical (diameter 30 mm, length 70 mm).

The finite element analysis was done with the simulation module of the Solid Edge software. The simulation conditions for all simulations performed were: mesh type—tetrahedral; study—linear static; meshing level—9. For this meshing level the mesh size is between 1.51 and 3.45 mm. This value depends on the modelling type, dimensions and shapes of the specimens.

## 4. Results and Discussion

**Figure 4:**
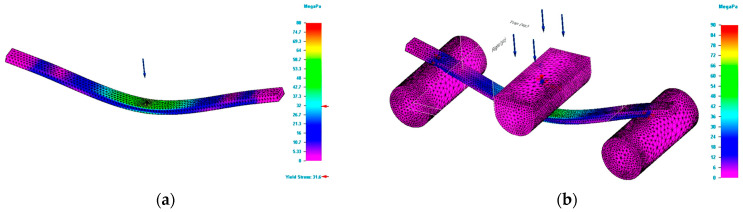
Result of simulation process for G_R5 specimen (Von Mises stress). (**a**) Von Mises stress for simple modelling. (**b**) Von Mises stress for complex modelling.

The bending tests carried out were aimed at determining the force and the deflection at which the tested specimens break. The value of the force allows calculation of the bending strength. The force determined for each specimen together with the dimensions of the specimen and the distance between the supports are the data that permit the bending strength to be calculated. Moreover, the mentioned information together with the mechanical properties of the 3D printed specimens were the input data for the bending test simulation process. The bending simulation was done based on both simple and complex modelling. Figure 4 shows the values of Von Mises stresses obtained by simulation for specimens having the rectangular cross section and dimensions of 5 × 10 mm.

In Figure 4b, the supporting pin and loading pin are drawn at natural scale. For Figure 4a, the mentioned parts are drawn as schematic (figurative) forms. It can be seen that for the most unfavourable area located in the middle (centre) of the specimen, the values of the stresses for the two studied schematizations are close to the value established in the tests. Table 2 presents the results of the test and simulations process for the bending study of the 3D printed poly(lactic acid) structures.

**Figure 5:**
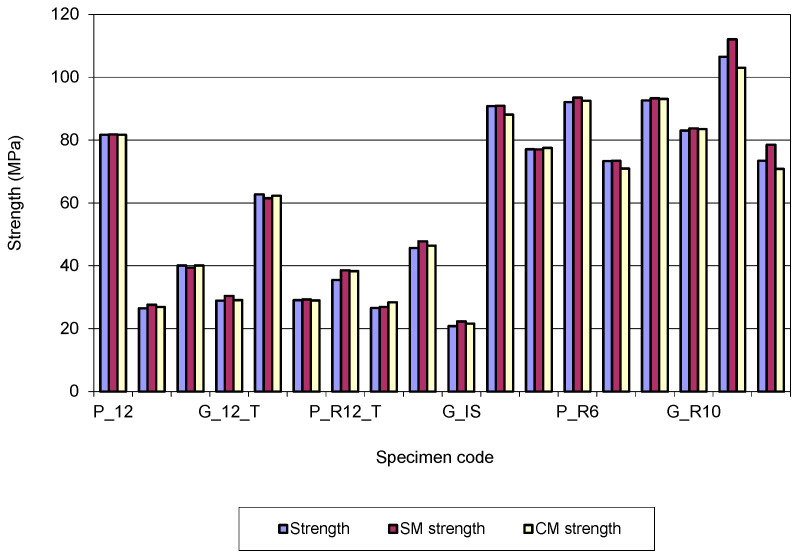
Evolution of the bending strength values for 3D printed specimens.

Moreover, in Figure 5 below, which has been graphed for an easier understanding of the data in Table 2 above, it can be seen that for most of the specimens studied, the complex modelling gives values almost identical to those recorded in the tests.

In other words, the results obtained by simulation for the Von Mises stresses based on the complex schematization are closer to the tests than those obtained based on the simple schematization. The study of Table 2 and Figure 5 shows that the bar-type specimens have the greatest values for the strengths. Regarding the efficiency of the material consumption, the most favourable results for the strength are for the tube-type specimens or I-section. For the bar-type specimens with the rectangular cross section, the strength has great values for those with the slender cross section (height greater than width).

**Figure 6:**
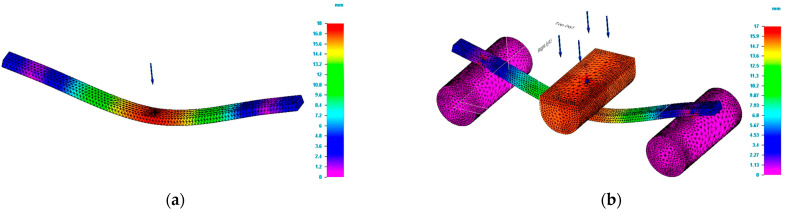
Result of simulation process for G_R5 specimen (total deformation values). (**a**) Total deformation values for simple modelling. (**b**) Total deformation values for complex modelling.

**Figure 7:**
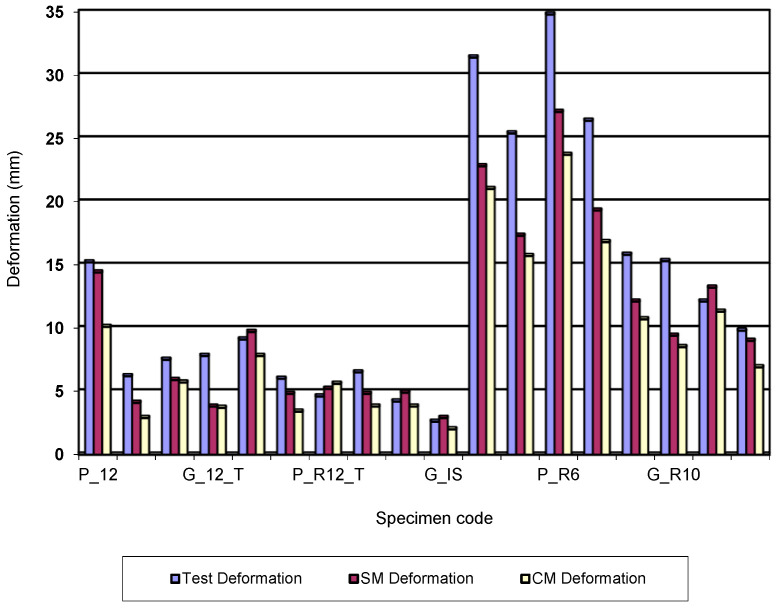
Evolution of the bending total deformation values for 3D printed specimens.

Figure 6 shows the simulation of deformations (deflexion) for specimens having the rectangular cross section and dimensions of 5 × 10 mm. The figures show that, in the case of complex modelling, the deformations determined by simulation have lower values than those determined in tests. Furthermore, the analysis of Figure 6 shows that the values of the deformations determined by simulation based on complex modelling are lower than those determined based on simple modelling. If the application of simulation in the case of Von Mises stresses leads to results close to those determined in tests, the same cannot be said for deformations. Although the complex modelling ensures a good replication (reproduction) of the bending behaviour, the application of the simulation process for deformations is not error-free. The analysis of Figure 7 shows that for bar specimens with a circular or rectangular cross section of bar or tube type, the deformations do not have high values and the differences between the values obtained in tests and those in simulation are not significant.

**Figure 8:**
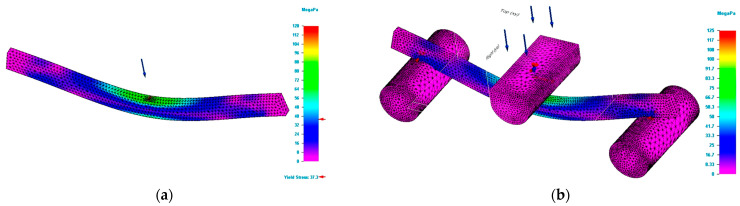
Result of simulation process for P_R15 specimen (Von Mises stress). (**a**) Von Mises stress for simple modelling. (**b**) Von Mises stress for complex modelling.

The explanation is due to the slender cross sections of the specimens, which do not allow large elastic deformations. In order to understand the cause of the errors, specimens with rectangular cross-sections of the same dimensions but arranged differently were additively manufactured. The specimens have dimensions (height x width) of 10 × 5 and 15 × 6 mm. These specimens were 3D printed under the same conditions (printing parameters were the same). Figure 8 shows the values of Von Mises stresses obtained by simulation for specimens having a rectangular cross section and dimensions of 15 × 6 mm.

**Figure 9:**
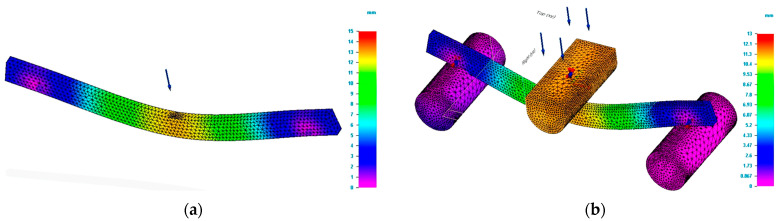
Result of simulation process for P_R15 specimen (total deformation values). (**a**) Total deformation values for simple modelling. (**b**) Total deformation values for complex modelling.

Figure 9 shows the simulation of deformations for specimens of rectangular cross section and dimensions of 15 × 6 mm.

Figure 7 also shows that for the last eight specimens, the differences between the deformations recorded in tests and those obtained by simulation are larger. Moreover, there are significant differences between the results of the complex schematic and those of the simple schematic.

Analysis of the stress-strain diagram plotted during the bending test allows the modulus of elasticity to be determined. The modulus of elasticity, being a property of the material, should have the same value for the two types of specimens under consideration (rectangular 5 × 10 mm, 6 × 15 mm, respectively 10 × 5 mm and 15 × 6 mm). The study of the elastic deformations determined in the tests shows that for the 3D printed PLA specimens, the values are 23 and 24 mm (code P_R5 and P_R6, respectively). For the same property, the values are 11 mm for P_R10 specimens and 9 mm for P_R15 specimens. For poly(lactic acid) specimens mixed with glass fibres, the elastic deformations are 17 mm for G_R5, 19 mm for G_R6, 11 mm for G_R10 and 8 mm for G_R15. The values recorded in the tests are high, and they have an impact on the deformation values determined in the simulation process. These high values are the source of the errors recorded in the simulation process.

Regarding the bending behaviour of the 3D printed structures, Table 2 shows that the most effective cross section is the tubular one. The tubular section has a maximum efficiency of material consumption and deformations are reduced. Another cross section with good performance in terms of material consumption, stresses and deformations is the I-section.

The positive aspect of large elastic deformation is that it allows structures to be built to accept such behaviour without their integrity being destroyed. In practice, such demands are frequently encountered, and they can be met by using sections where the width is greater than the height. If, on the other hand, the structures should be rigid (have low deformations), then structures where the height is greater than the width can be used.

## 5. Conclusions

The paper presents the impact of bending test modelling on the results of the simulation process, applied to 3D printed specimens. Two types of filaments were used, one made of poly(lactic acid) and the second made of poly(lactic acid) mixed with glass fibres. For the considered schematizations, the complex one and the simple one, the analysis shows that in terms of Von Mises stresses determined by simulation, the values are close to those established in the tests. Moreover, it is found that in the case of the complex schematization, the simulation allows values to be obtained that are almost identical to those of the tests. The comparison between the two schematizations shows that for the Von Mises stresses determined by simulation, the results favour the complex schematization. From the point of view of the deformations determined by simulation, the schematizations used do not give values close to those recorded in the tests. In this case, the values close to those of the tests are provided by the simple schematization. Analysis of the simulation results for the bending of 3D printed structures shows that the use of the complex modelling offers a slight advantage compared to simple modelling, for Von Mises stresses. However, the time taken to perform complex schematization is much longer compared to the time taken to perform simple schematization.

The positive aspect of the study is that values of the total deformation established by simulation are lower than those obtained in tests. Thus, the tests show that additive manufactured structures, in reality, can be deformed more than the simulations indicate; therefore, there exists a reserve that allows the structures to be deformed without being destroyed. This behaviour is in connection with the value of the modulus of elasticity. The modulus of elasticity is influenced not only by printing parameters but also by the shape of the cross section, more precisely by the slenderness of the cross section.

Moreover, in order to minimize the errors that deformation simulation registers, additional attention should be given to the physical and technological properties of 3D printed structures, because their values represent the input data of the simulation process. These properties need to be carefully managed because the current study shows that they differ significantly from one cross-section geometry to another, although the printing parameters are the same. Additionally, the study shows that in the simulation process much attention must be given to the modulus of elasticity, since this value has a major influence on the deformation result.

## Figures and Tables

**Table 1:** Specimen characteristics and codification (coding).

Filament Type	Cross Section	Dimensions (mm)	Specimen Type	Supporting Span (mm)	Specimen Code
PLA	Circular	12	Bar	180	P_12
PLA-Glass	Circular	12	Bar	180	G_12
PLA	Circular	12 × 10	Tube	180	P_12_T
PLA-Glass	Circular	12 × 10	Tube	180	G_12_T
PLA	Rectangular	12.6 × 9	Bar	180	P_R12
PLA-Glass	Rectangular	12.6 × 9	Bar	180	G_R12
PLA	Rectangular	12.6 × 10.8	Tube	180	P_R12_T
PLA-Glass	Rectangular	12.6 × 10.8	Tube	180	G_R12_T
PLA	I-section	11.2 × 28	Bar	180	P_IS
PLA-Glass	I-section	11.2 × 28	Bar	180	G_IS
PLA	Rectangular	5 × 10	Bar	160	P_R5
PLA-Glass	Rectangular	5 × 10	Bar	160	G_R5
PLA	Rectangular	6 × 15	Bar	180	P_R6
PLA-Glass	Rectangular	6 × 15	Bar	180	G_R6
PLA	Rectangular	10 × 5	Bar	160	P_R10
PLA-Glass	Rectangular	10 × 5	Bar	160	G_R10
PLA	Rectangular	15 × 6	Bar	180	P_R15
PLA-Glass	Rectangular	15 × 6	Bar	180	G_R15

**Table 2:** Comparison between results of the tests and simulation, for 3D printed specimens, bending stressed.

Specimen Code	Test Results		SM Simulation Results		CM Simulation Results	
	Strength (MPa)	Deformation (mm)	Strength (MPa)	Deformation (mm)	Strength (MPa)	Deformation (mm)
P_12	81.70	15.20	81.80	14.40	81.70	10.10
G_12	26.50	6.20	27.60	4.10	26.90	2.90
P_12_T	40.10	7.50	39.40	5.90	40.10	5.70
G_12_T	28.90	7.80	30.40	3.80	29.10	3.70
P_R12	62.70	9.10	61.50	9.70	62.30	7.80
G_R12	29.10	6.00	29.30	4.80	29.00	3.40
P_R12_T	35.50	4.60	38.60	5.20	38.30	5.60
G_R12_T	26.60	6.50	28.90	4.80	28.40	3.80
P_IS	45.70	4.20	47.80	4.90	46.40	3.80
G_IS	20.80	2.60	22.30	2.90	21.60	2.00
P_R5	90.80	31.40	90.90	22.80	88.10	21.00
G_R5	77.10	25.40	77.00	17.30	77.50	15.70
P_R6	92.10	34.80	93.50	27.10	92.50	23.70
G_R6	73.30	26.40	73.40	19.30	70.90	16.80
P_R10	92.60	15.80	93.30	12.10	93.10	10.70
G_R10	83.00	15.30	83.70	9.40	83.50	8.50
P_R15	107	12.10	112	13.20	103	11.30
G_R15	73.40	9.80	78.50	9.00	70.80	6.90

## Data Availability

Not applicable.
